# Methamphetamine mediates immune dysregulation in a murine model of chronic viral infection

**DOI:** 10.3389/fmicb.2015.00793

**Published:** 2015-08-11

**Authors:** Uma Sriram, Bijayesh Haldar, Jonathan M. Cenna, Larisa Gofman, Raghava Potula

**Affiliations:** ^1^Department of Pathology and Laboratory Medicine, Temple University School of MedicinePhiladelphia, PA, USA; ^2^Center for Substance Abuse Research, Temple University School of MedicinePhiladelphia, PA, USA

**Keywords:** drug abuse, immune response, T cells, viral infection, LCMV

## Abstract

Methamphetamine (METH) is a highly addictive psychostimulant that not only affects the brain and cognitive functions but also greatly impacts the host immune system, rendering the body susceptible to infections and exacerbating the severity of disease. Although there is gathering evidence about METH abuse and increased incidence of HIV and other viral infections, not much is known about the effects on the immune system in a chronic viral infection setting. We have used the lymphocytic choriomeningitis virus (LCMV) chronic mouse model of viral infection in a chronic METH environment and demonstrate that METH significantly increases CD3 marker on splenocytes and programmed death-1 (PD-1) expression on T cells, a cell surface signaling molecule known to inhibit T cell function and cause exhaustion in a lymphoid organ. Many of these METH effects were more pronounced during early stage of infection, which are gradually attenuated during later stages of infection. An essential cytokine for T-lymphocyte homeostasis, Interleukin-2 (IL-2) in serum was prominently reduced in METH-exposed infected mice. In addition, the serum pro-inflammatory (TNF, IL12 p70, IL1β, IL-6, and KC-GRO) and Th2 (IL-2, IL-10, and IL-4) cytokine profiles were also altered in the presence of METH. Interestingly CXCR3, an inflammatory chemokine receptor, showed significant increase in the METH treated LCMV infected mice. Similarly, compared to only infected mice, epidermal growth factor receptor (EGFR) in METH exposed LCMV infected mice were up regulated. Collectively, our data suggest that METH alters systemic, peripheral immune responses and modulates key markers on T cells involved in pathogenesis of chronic viral infection.

## Introduction

Methamphetamine (METH) is an extremely addictive central nervous system (CNS) stimulant. Recreational METH use is one of the fastest growing substance abuse problems in the United States (Cruickshank and Dyer, 2009). METH is self-administered intravenously, by nasal inhalation, anally, and orally, in doses of 250–500 mg by occasional users to as much as 1 g by chronic abusers (Tallóczy et al., 2008). Although there is substantial evidence of the effects of METH on CNS functions (Loftis and Janowsky, 2014), the effects of METH on systemic immune responses has not been extensively described. Few studies that have addressed this demonstrated that METH suppresses both innate and adaptive immunity in animals and humans (Yu et al., 2002; Martinez et al., 2009; Harms et al., 2012). These and other studies reveal that METH profoundly interferes with immunological networks and affects diverse leukocyte subsets, thereby increasing susceptibility to infection (Ellis et al., 2003; Martinez et al., 2009). *In vitro* use of amphetamines, including METH, affects immune function with a significant suppression of IL-2 (Potula et al., 2010), but not IL-4 production by T-lymphocytes, as well as a suppression of B-lymphocyte proliferation; however, this occurred only at the highest amphetamine concentrations (Steinkellner et al., 2011; Kwack et al., 2014).

Considerable evidence exists linking drug abuse to immune dysregulation and enhanced susceptibility to the progression of chronic infections, such as HIV-1(Ellis et al., 2003; Mantri et al., 2014). METH use is associated with high-risk sexual behavior and high rates of HIV acquisition and progression (Jamieson et al., 1997; Ellis et al., 2003). In this report, we have used the mouse model of chronic lymphocytic choriomeningitis virus (LCMV) infection to study the effects of METH on T cell immune responses. Although LCMV is a relatively simple virus, encoding only four gene products, it has proven to be one of the best experimental systems for analyzing cellular immune responses (Zhou et al., 2012). Several studies have reported that acute infections induce remarkably high levels of antiviral T cells, while protracted or chronic infections are associated with both functional impairment and deletion of virus-specific CD8 T cells (Khanolkar et al., 2002). T cell exhaustion has a major role in failure to control chronic infection. Expression of inhibitory receptors, including PD-1, an inhibitory receptor of the CD28–CTLA-4 family are up regulated considerably in chronic viral infection (Barber et al., 2006). This along with the inability to sustain functional T cell responses contribute to exhaustion. CD4 Th cells are central orchestrators of the immune response and differentially activate diverse branches of innate and adaptive immunity to guide the appropriate response to an invading pathogen (Penaloza-MacMaster et al., 2014). CD4 Th1 immunity is critical to sustain residual CD8 T-cell activity to control infection during persistent infection and is characterized in CD4 T cells by the secretion of IFN-γ, TNF-α, and IL-2 (Matloubian et al., 1994).

So far no study has addressed the role of METH in the context of chronic viral infection to analyze the effects on T cell immune responses. In this report, we have systematically analyzed the classic responses of CD4 and CD8 T cells in secondary lymphoid organ namely spleen during chronic LCMV infection in mice that have been exposed to chronic METH and the peripheral responses by measuring the serum cytokines.

Our findings indicate that METH administered in a s.c. route altered T cells responses with important consequences, in a chronic LCMV infection model. METH effects on CD4 and CD8 cell percentages *per se* were modest although the expression of important markers of LCMV infection and T cell exhaustion such as PD-1 was greatly increased. Many of the METH effects were more pronounced by day 14 but normalized as infection progressed up to 56 days. Serum cytokine analysis revealed reduction of IL-2 production at all time points in METH-exposed infected mice than without. The serum pro-inflammatory (TNF, IL12p70, IL1β, IL-6, and KC-GRO) and Th2 (IL-2, IL-10, and IL-4) cytokine profiles were also altered in the presence of METH. Interestingly CXCR3, an inflammatory chemokine receptor, showed significant increase in the METH treated LCMV infected mice, suggesting that METH modulates the migratory properties of T cells during infection thus affecting immune activation. We also found another interesting up regulation of epidermal growth factor receptor (EGFR) in METH exposed LCMV infected mice at later times of infection, suggesting that signaling through EGFR may enable to establish persistent infection.

## Materials and methods

### Mice

Male C57BL/6 mice 4 weeks of age were purchased from Jackson Labs, housed in specific pathogen free conditions, and given unlimited access to food and water. Protocols for the use of animals were in accordance with the guidelines of and were approved by the Institutional Animal Care and Use Committees of Temple University, which is American Association for the Accreditation of Laboratory Animal Care accredited facility.

### METH treatment and LCMV infection

**Figure 1:**
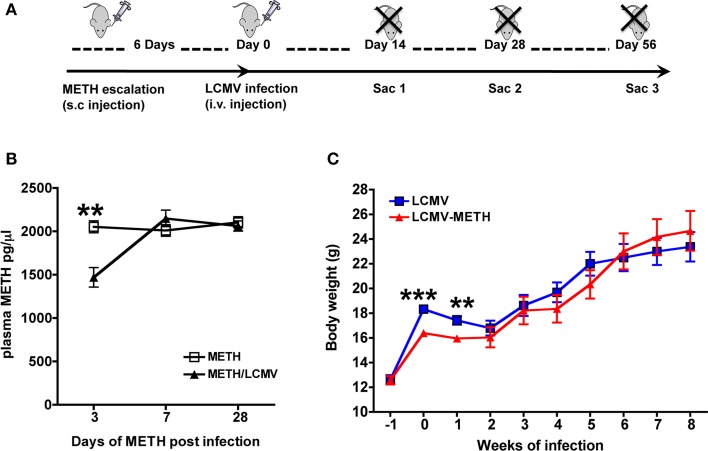
**METH levels in plasma during chronic exposure and LCMV infection. (A)** Experimental scheme of METH administration and LCMV infection. **(B)** ELISA was performed to measure steady-state METH levels after the different time-points indicated. Results are expressed as Mean ± SE of at least 5 measurements from each group at each time-point. METH vs. LCMV-METH ^**^p < 0.01 at day 3. **(C)** Measurement of body weight after METH treatment in LCMV infected mice. Body weights of mice before and after METH administration and infection were measured and expressed as grams (Mean ± SE of at least 10 mice per group). Body weights significantly decreased with METH at very early infection ^**^p < 0.01 and ^***^p < 0.001.

Methamphamphetamine Hydrochloride was purchased from Sigma-Aldrich (St. Louis, MO). Mice were weight-matched and randomly divided into groups and were administered a gradual escalating METH dose from 0.45–10 mg/kg over 6 days, followed by a single dose of 10 mg/kg/day subcutaneously (s.c. at the nape of the neck) until the mice were sacrificed. Once daily injection of METH was established, mice were intravenously injected with LCMV Clone 13 (2 × 10^6^ pfu). Experimental scheme is shown in Figure 1A. Peripheral blood was collected via submandibular vein into EDTA coated collection tubes (BD Biosciences) every 7 days until the end of the experiment. METH levels in plasma were assayed using a sensitive Elisa kit from Abnova (Taipei City, Taiwan). To determine the steady-state levels of METH upon daily injection, mice were bled at the same time at every time-point, to eliminate bias in the half-life of METH. Blood was collected after 2 h of METH injection at each time-point.

### Quantitation of viral load

LCMV viral RNA was extracted from harvested and cleared plasma, lung and spleen samples using Viral RNA extraction kit (Qiagen). Extracted RNA was cleared of any genomic DNA contamination using the DNA-free kit (Ambion). Complimentary DNA was then generated using Superscript III Reverse Transcriptase (Life Sciences) utilizing 10 μl of RNA template and forward and reverse primers designed specifically for the LCMV glycoprotein (GP). Real-time quantitative PCR of LCMV viral cDNA was performed using LCMV GP primers and Sybr Green Master Mix (Fermentas and analyzed using StepOne Plus Real Time PCR system. Resulting C_T_-values were used measured against a standard curve generated from the LCMV-Arm53b plasmid (gift of Addgene plasmid #15796) of known copy numbers to calculate average log viral copy numbers/mL of plasma (Pal et al., 2014). Samples and standards were performed in triplicate with five animals per experimental condition.

### Flow cytometry

Splenocytes extracted from LCMV infected mice were subjected to FACS analysis on a FACS Canto II (BD Biosciences). The lymphocyte population was gated based on forward and side scatter. T lymphocyte population was gated using CD3 and further divided as CD4+ and CD8+ subsets. After washing, splenocytes were resuspended in R10 media containing LCMV Peptide 69 (Proimmune, epitope of GP1, 1 μg/ml) and Brefeldin A (Ebioscience, 1 μl/ml). After incubation for 5–6 h at 37°C, cells were centrifuged, washed and then stained with PE labeled Pro5® MHC class I Pentamer (Proimmune, 10 μl/test) specific to allele H-2Db and other fluorescence conjugated antibodies to cell surface markers and intracellular cytokines for flow cytometric analysis. The following antibodies were used for staining: CD3 APC-Cy7, CD4 AmCyan, CD8 Pacific Blue, LCMV Pentamer PE, CD279 (PD1) PECy7, CD25 PerCpCy5.5, CD69 APC, CD27 PeCy7, CXCR3 PerCpCy5.5, and EGFR FITC (antibodies were from eBiosciences, San Diego, and BD Biosciences, CA, USA). Stained cells were then acquired on a FACS Canto II (BD Biosciences) using FACS Diva software (BD Biosciences, V 6.1.3). Cells were gated using isotype control as negatives (Figure S1 in Supplementary Material). A representative plot for the gating strategy for CD3+, CD4+, and CD8+ is shown in **Figure 3A**. At least 10,000 lymphocyte events were recorded. Recorded data was plotted and further analyzed using FlowJo software (TreeStar V 9.4). Values from FlowJo were transferred into GraphPad Prism (v. 4.0, GraphPad Software) for statistical analysis.

### Serum cytokine analysis

Mouse blood was collected from submandibular vein at different time points and serum was separated. Multiplex mouse cytokine analysis was performed using Meso-scale Discovery® (MSD) multi-array system as per manufacturer's protocol. Briefly 25 μl of serum was applied into MSD ELISA, and cytokine levels were estimated using provided standards and calculated by MSD reader software (Meso Scale Discoveries, Rockville, MD, USA).

### Statistical analysis

Data were analyzed using Prism software (GraphPad, San Diego, CA). One sample *t*-test, two-tailed Student's *t*-test or One-Way ANOVA, as appropriate, was used as statistical test for different sets of experiments, and considered significant values at *p* < 0.05 (marked in the figures as ^*^p < 0.05; ^**^p < 0.01; ^***^p < 0.001).

## Results

### METH levels in plasma during chronic exposure and LCMV infection

We analyzed METH levels in the plasma at different time points after LCMV infection, to determine if levels of METH were affected during viral infection. In our model, chronic exposure of 10 mg per day METH was well-tolerated up to 56 days (data not shown). Steady-state plasma METH levels were analyzed up to 28 days (Figure 1B). Plasma METH levels was analyzed 2 h after METH injection at each time-point and the levels were steadily at about 2000 pg/μl and sustained for up to 28 days. Several studies have reported the levels of METH in plasma and other organs in human, murine and rodent species (Riviére et al., 2000; Volkow et al., 2010; Loftis et al., 2011) after various routes of injection. Our analysis via s.c. route also show that steady-state METH levels in circulation is close to what has been reported in these studies between 1000 and 2000 pg/μl after about 2 h of METH administration (Riviére et al., 2000). Interestingly, plasma METH levels were significantly lower in the LCMV infected mice at day 3 as compared to METH alone but quickly normalized and remained unaltered between the two groups up to 28 days of analysis (in pg/μl, METH—2053 ± 68.0 vs. METH-LCMV—1470 ± 113) (Figure 1B). METH is mainly metabolized in the liver and the LCMV virus (clone 13) also has been shown to infiltrate the liver by the first week of infection (Beura et al., 2015). One plausible speculation could be that high viremia during early stage of infection might have an effect on the metabolism of METH in the liver and release in circulation. In addition, hepatic and renal clearance also contribute to the elimination of METH. Quantitative excretion profile of METH and its levels in organs could help further address the differences. While the body weights were lower in the initial two weeks of LCMV infection in the METH treated group (LCMV—17.4 ± 0.4 g vs. LCMV-METH—16.0 ± 0.3 g), for the rest of the time up to 8 weeks there was no difference in the body weights between the METH treated LCMV infected mice and LCMV alone (Figure 1C).

### METH treatment does not alter plasma or organ viral load in chronic persistent LCMV infection

**Figure 2:**
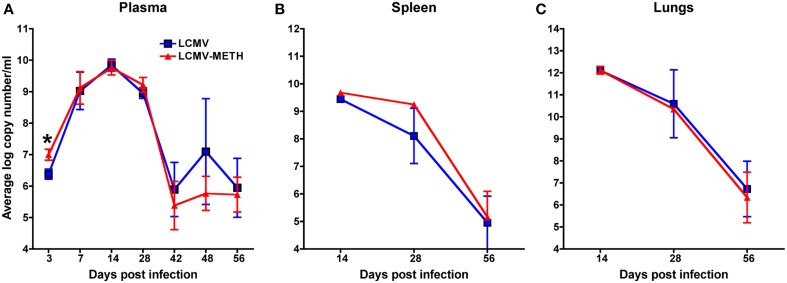
**METH treatment does not alter plasma or organ viral load in chronic LCMV infection**. LCMV viral load in plasma **(A)**, spleen **(B)**, and lungs **(C)** was measured by RT-PCR. Real-time quantitative PCR of LCMV viral cDNA was performed using LCMV GP primers and Sybr Green Master Mix. A standard curve was generated from the LCMV-Arm53b plasmid to calculate the copy numbers. Samples and standards were performed in triplicate with five animals per experimental condition.

We analyzed the LCMV viral load in circulation (plasma—Figure 2A) and also in the organs (spleen and lungs—Figures 2B,C, respectively) by quantitative PCR after chronic METH exposure. There was a small but significant increase in the viral load in the plasma at day 3 in METH group than without (LCMV—6.4 ± 0.2 vs. LCMV-METH—7.0 ± 0.2 average log copy numbers/ml; *n* = 24 in each group; *p* < 0.05), that quickly normalized as the infection proceeded (Figure 2) suggesting METH affects viral infection at very early stages of viral replication. Our observation is in concordance with Bourne's group (Valencia et al., 2012) that showed similar increase in Herpes simplex virus upon METH treatment by day three post-infection, in a mouse model. Plasma, spleen, and lungs showed similar viral titer by day 14. However, the viral loads with or without METH at any later time points, either in plasma or in the organs were not different (Figure 2) indicating that chronic exposure did not affect the viral replication throughout the time of the study. Similar observations have been made in the herpes simplex virus model that is studied in the presence of METH as well (Valencia et al., 2012). Viral load peaked at day 14 and decreased steeply between day 28 and 56 in plasma, spleen and lungs, in both the infected mice with or without METH. This observation is similar to the trend in chronic infection observed in other studies (Matloubian et al., 1994). In mice, LCMV viral replication in most tissues is controlled by 3 months post-infection, but the virus persists in some tissues for life (Ahmed and Oldstone, 1988; Wherry et al., 2003; Kao et al., 2011).

### METH treatment significantly increases total CD3+ but slightly alters percentages of CD4+ and CD8+ T cell subsets in spleen at early stages of chronic LCMV infection

**Figure 3:**
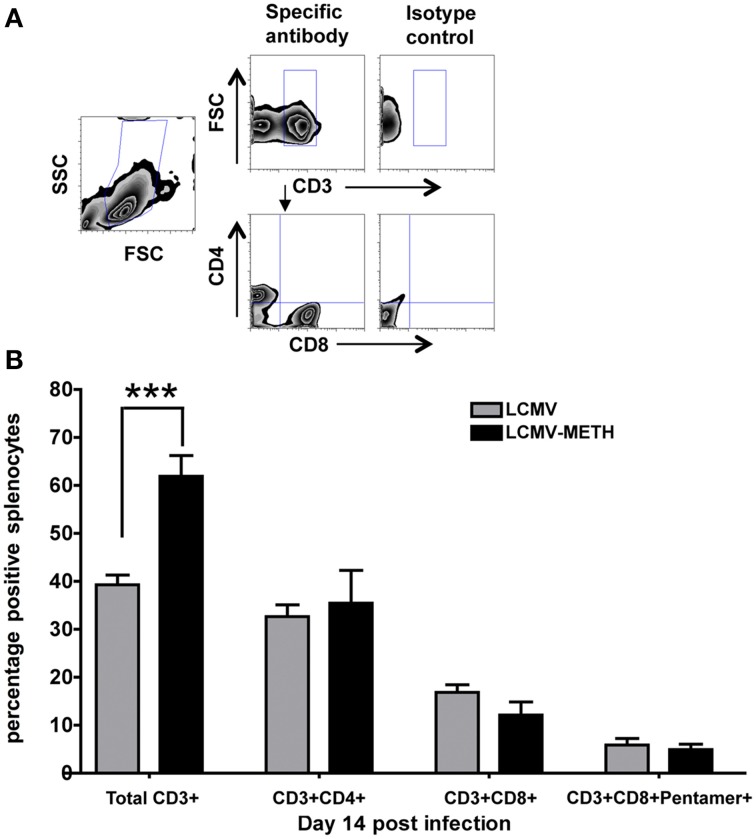
**METH treatment alters percentages of CD3+ but not CD4+ and CD8+ T cells in spleen at early stages of chronic LCMV infection**. Splenocytes extracted from LCMV infected mice with or without METH exposure, were surface stained with CD3, CD4, CD8, and Pentamer specific CD8+, markers after antigen specific stimulation against Peptide 69. **(A)** Gating strategy for flow cytometric analysis. **(B)** Percentages of CD3+, CD4+, CD8+, and Pentamer specific T cell populations are shown as Mean ± SE of at least 8 mice per group. CD3 surface marker was significantly increased in the METH treated LCMV infected group than LCMV alone; ^***^p < 0.001.

METH administration affects a vast repertoire of immune cells including natural killer cells, dendritic cells, monocytes, macrophages, and granulocytes (Harms et al., 2012). T cells play a critical role in orchestrating immune responses and METH has been shown to adversely impact T cell responses, especially in HIV infection (In et al., 2005; Martinez et al., 2009). Chronic LCMV infection in mice causes huge alterations in the T cell immune subsets in the spleen (Khanolkar et al., 2002). To study if chronic METH exposure altered these changes in the CD4+ and CD8+ T cell compartments, we analyzed changes in total CD4+ and CD8+ T cells as well as antigen specific CD8+ subsets using Pentamer staining. A representative plot for the gating strategy for CD3+, CD4+, and CD8+ is shown in Figure 3A. To our surprise, we did not see any marked differences in the CD4 and CD8 T cell populations or the percentage of antigen specific CD8+ T cells, between METH treated infected mice and infected alone (Figure 3B). It has to be noted that the total CD4+ cells did not change much during the course of infection while CD8+ T cells increased significantly by day 56 (Mean percent ± SE–CD4+ LCMV–day 14–32.7 ± 2.4; day 28–44.0 ± 3.1; day 56–41.7 ± 2.7; CD8+ LCMV–day 14–17.2 ± 1.6; day 28–22.2 ± 4.8, day 56–28.9 ± 3.9, day 14 vs. day 56 ^*^p < 0.01), and METH did not modulate the CD4+ or CD8+ expression at later time points as well (data not shown). We did see an increased percentage of CD8+ T cells upon METH treatment as compared to no METH controls in uninfected mice (data not shown) at day 14. We also saw a significant increase in the total CD3+ T cells at day 14 upon METH treatment in infected mice (LCMV—39.3 ± 2.0 % vs. LCMV-METH—61.9 ± 4.3%; ^***^p < 0.001—Figure 3B). The increase in CD3+ cells upon METH in the infected group may suggest that these cells may be activated. Peerzada et al. have shown a strong decrease in the splenic CD3+ cells upon METH treatment (Peerzada et al., 2013) that we did not observe in our uninfected METH /no METH study groups (data not shown).

### METH induces significant increase in PD-1 in the splenic CD4+ and CD8+ T cells at early stages of chronic LCMV infection

**Figure 4:**
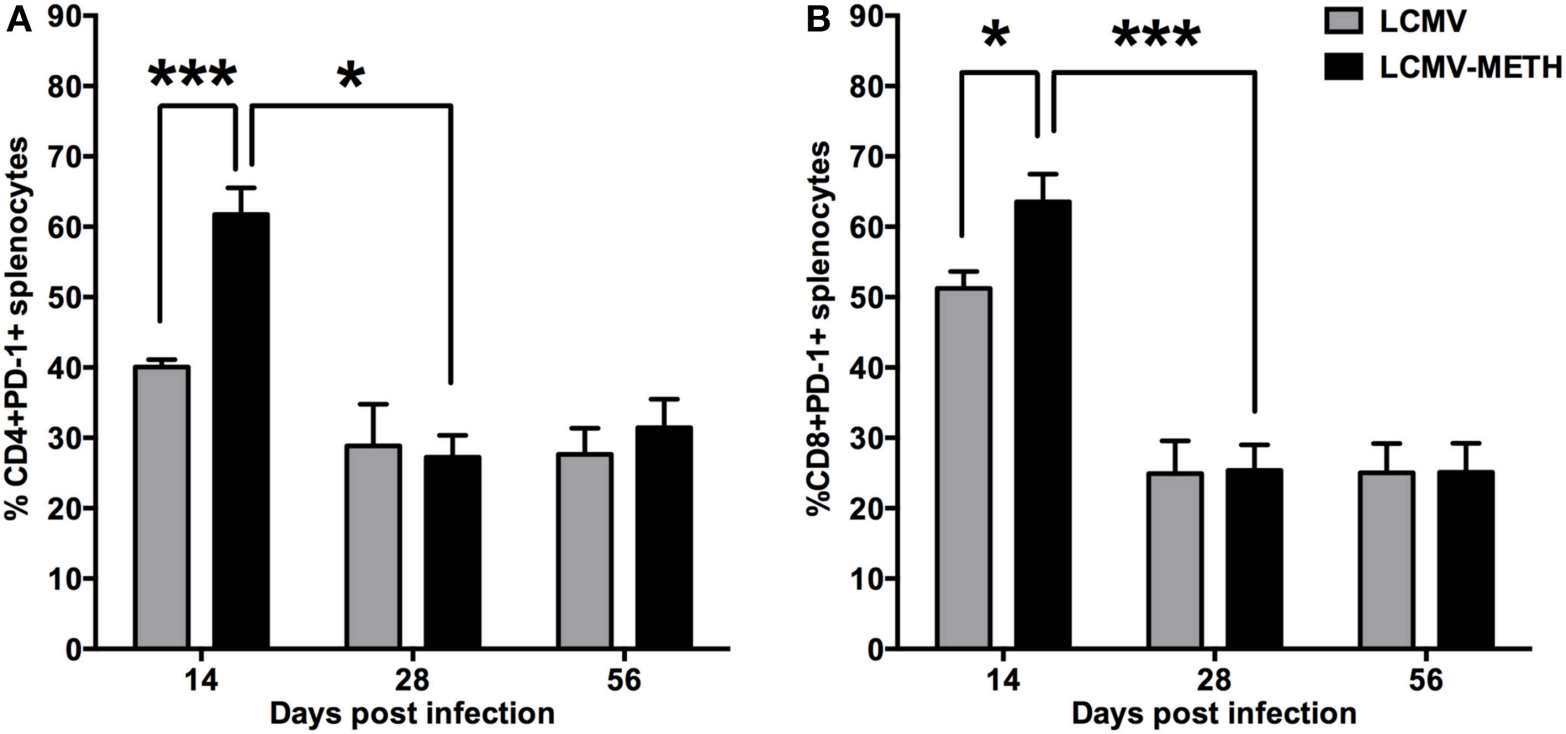
**METH induces significant increase in PD-1 in the splenic CD4+ and CD8+ T cells at early stages of chronic LCMV infection**. Splenocytes extracted from LCMV infected mice with or without METH treatment, were surface stained after antigen specific stimulation, with CD3, CD8, CD4 and PD-1(CD279) markers to analyze the classic activation with LCMV infection. FACS analysis was performed and analyzed as described in Methods section. METH significantly increased PD-1 expression by day 14 of infection both in CD8 (^*^p < 0.05) **(B)** and very significantly in the CD4 (^***^p < 0.001) **(A)** T cells.

CD8 T cells undergo depletion or functional inactivation also known as exhaustion in chronic viral infection. The functional exhaustion of CD8 T cells has been shown to correlate with PD-1 expression (Blattman et al., 2009; Kao et al., 2011; Penaloza-MacMaster et al., 2014) and the first studies to demonstrate this comes from LCMV chronic mouse model (Barber et al., 2006). Therefore we analyzed the expression of this important inhibitory marker in a chronic METH environment. We hypothesized that chronic METH exposure augments the expression of the inhibitory receptor PD-1 in the LCMV chronic infection model and indeed we saw a significantly increased PD-1 expression in the METH treated infected group at day 14 both in the CD4+ and CD8+ T cells than the non-METH infected mice (Figure 4). However, the effect with METH treatment was seen only at day 14 and normalized as the infection progressed (Figure 4). There was a significant decrease in the CD4+ and CD8+ cells expressing PD-1 by day 28 after which the expression plateaued up to day 56 (LCMV—CD4+PD-1+—day 14 vs. 28—^*^p < 0.05; CD8+PD-1+—day 14 vs. day 28—^***^p < 0.002—Figure 4). Presence of METH did not change the expression of PD-1 at these time-points probably because the cells were already functionally exhausted. PD-1 increase by METH suggests that chronic METH exposure might augment CD8+ T cells exhaustion thus making them functionally inactive to evoke any anti-viral immunity. CD4+ T cell help is essential to raise and maintain an efficient CD8 response (Matloubian et al., 1994). Increase in PD-1 in the CD4+ T cells therefore suggests that the CD4+ T cells also undergo exhaustion in the presence of METH and the required CD4+ T cell help to evoke CD8+ responses is diminished as well (Matloubian et al., 1994; Han et al., 2010).

### METH modulates CD69, CD25, and CD27 activation markers in the splenic T cell subsets in chronic LCMV infection

One of the earliest cell surface antigens expressed by T cells following activation is CD69, a member of the C-type lectin superfamily (Marzio et al., 1999). Once expressed, CD69 acts as a costimulatory molecule for T cell activation and proliferation. We analyzed the expression of CD69 in the context of LCMV in a METH environment. There was a significant increase in CD69 marker on CD8 T cells at day 14 (*p* < 0.05) (Table 1) while METH did not induce differential expression of this marker at other time points. Although not statistically significant a similar trend of increased CD69 expression was noted on CD4+ T cells at day 14 and day 28 post-infection (Table 1). The expression of CD69 significantly decreased during the course of infection (CD4–LCMV—day 14 vs. day 56—^**^p < 0.01; CD8-LCMV—day 14 vs. day 56—^*^p < 0.05). The induction of CD69 by METH at early time-point during viral infection might play an important role in regulation of the subsequent immune responses.

**Table 1:** **Modulation of activation markers on CD4+ and CD8+ T cells induced upon LCMV infection by chronic METH exposure**.

**Activation marker**	**T cell subset**	**Treatment**	**Days post infection**		
			**14**	**28**	**56**
CD69	CD4	LCMV	36.8 ± 3.1	23.1 ± 2.8	17.1 ± 3.6^**^
		LCMV-METH	46.9 ± 8.1	26.7 ± 3.7	17.2 ± 1.8^**^
	CD8	LCMV	19.5 ± 2.2	21.2 ± 4.0	11.5 ± 2.7^**^
		LCMV-METH	33.1 ± 8.5^*^	23.7 ± 4.4	9.1 ± 2.1^*^
CD25	CD4	LCMV	35.5 ± 7.2	24.4 ± 9.4	18.5 ± 4.0
		LCMV-METH	29.6 ± 3.1	22.3 ± 5.4	29.6 ± 7.2
	CD8	LCMV	5.8 ± 1.1	5.1 ± 2.8	6.5 ± 1.9
		LCMV-METH	6.8 ± 0.9	4.4 ± 0.9	4.7 ± 1.0
CD27	CD4	LCMV	8.4 ± 2.0	5.6 ± 1.7	3.7 ± 0.6
		LCMV-METH	5.9 ± 1.3	4.8 ± 1.0	4.7 ± 1.0
	CD8	LCMV	8.2 ± 1.8	6.2 ± 1.7	5.5 ± 1.4
		LCMV-METH	7.8 ± 1.5	5.8 ± 1.0	5.0 ± 1.3

Splenocytes extracted from LCMV infected mice, with or without METH treatment, were surface stained after antigen specific stimulation for (1) T cell markers: CD3, CD8, and CD4 and (2) activation markers: CD69, CD25, and CD27. FACS analysis was performed and analyzed as described in Materials and Methods Section. Numbers are percent positive cells for the respective marker. METH significantly increased the CD69 marker on CD8 T cells of the LCMV-METH group vs. LCMV on day 14 (*p < 0.05). CD69 was significantly decreased over the course of infection; d14 vs. d56–

** p < 0.01 for CD4 and

* *p < 0.05 for CD8 subsets. METH did not significantly alter the expression of either CD25 or CD27 at any time point analyzed*.

CD25 is the alpha chain of the IL-2 receptor. The CD4+CD25+ subset alone or along with the expression of FoxP3 is often referred to as T suppressor/T regulatory subset (Hori et al., 2003). We analyzed the modulation of CD25 in both CD4 and CD8 T cell compartments. The percentages of CD4+CD25+ were only slightly altered by METH and did not change much in the CD8 compartment as well as in the infected groups (Table 1). METH did not alter the expression of CD25 in CD4+ or CD8+ cells without infection as well (data not shown).

CD27 is a member of Tumor necrosis factor receptor (TNFR) family members that play key roles in control of both acute and persistent or latent infections (Welten et al., 2013). CD27 is constitutively expressed on naive T cells. Analysis of this marker in the LCMV infected group with or without METH showed slight decrease in the CD27 expression at day 14 in the METH than the no METH CD4+ subset (Table 1), but no significant alterations in the percentages of this marker on CD4+ or CD8+ cells at any other later time points analyzed. It has been shown that during acute (Armstrong) or chronic LCMV (clone 13) infection, CD27 initially increases and then returns to the baseline levels with T cells remaining CD27-positive throughout the infection (Welten et al., 2013; Mbanwi and Watts, 2014). Our observation in the LCMV infected group is similar to these reports. However, our results suggest that METH may not affect expression of this marker on splenic T cells.

Overall, none of the markers of classic T cell activation was greatly modulated by METH during LCMV infection, suggesting that METH did not directly affect T cell activation via the expression of these markers but may alter T cell function via cytokine production that may be mediated by other pathways.

### CXCR3 expression on splenic T cell subsets is exacerbated in METH administered LCMV mice

**Figure 5:**
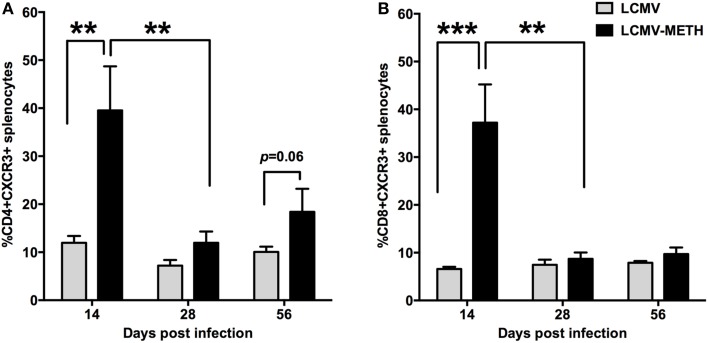
**CXCR3 expression on splenic T cell subsets is exacerbated in METH administered LCMV mice**. Splenocytes extracted from LCMV infected mice with or without METH treatment, were surface stained after antigen specific stimulation with CD3, CD8, CD4 and CXCR3 markers. FACS analysis was performed and analyzed as described in Methods section. METH significantly increased CXCR3 expression by day 14 of infection both in CD4 (^**^p < 0.01) **(A)** and CD8 (^***^p < 0.001) **(B)** T cells and persistent increase was seen in CD4 T cells at later time-points as well when compared to no METH LCMV infected group. However, the expression levels induced by METH significantly dropped from day 14 during the course of infecion (^**^p < 0.01), while infection *per se* without METH did not alter CXCR3 expression levels.

CD8 T cells alter their expression of chemokine receptors upon activation by both antigen and innate stimuli (Christensen et al., 2006; Kroll et al., 2014; Torraca et al., 2015). It has been shown that inflammatory chemokine receptors, such as CXCR3, CXCR6, and CCR5, are upregulated during infection. We analyzed in our model if METH altered CXCR3 expression and found that CXCR3 expression on both CD4+ and CD8+ T cells was very significantly upregulated in the METH treated infected group than LCMV alone by day 14, and this increase although not significant was persistent up to 56 days in CD4+ (Figure 5A) but not CD8+ T cells (Figure 5B). Alteration in CXCR3 expression has been shown to affect induction of central and effector memory T cell responses (Hu et al., 2011). Our current findings of increased CXCR3 suggest an important role of METH in altering the recruitment of T cells and probably generation of memory response.

### Chronic METH treatment increases EGFR expression on splenic T cells subsets in LCMV infection

**Figure 6:**
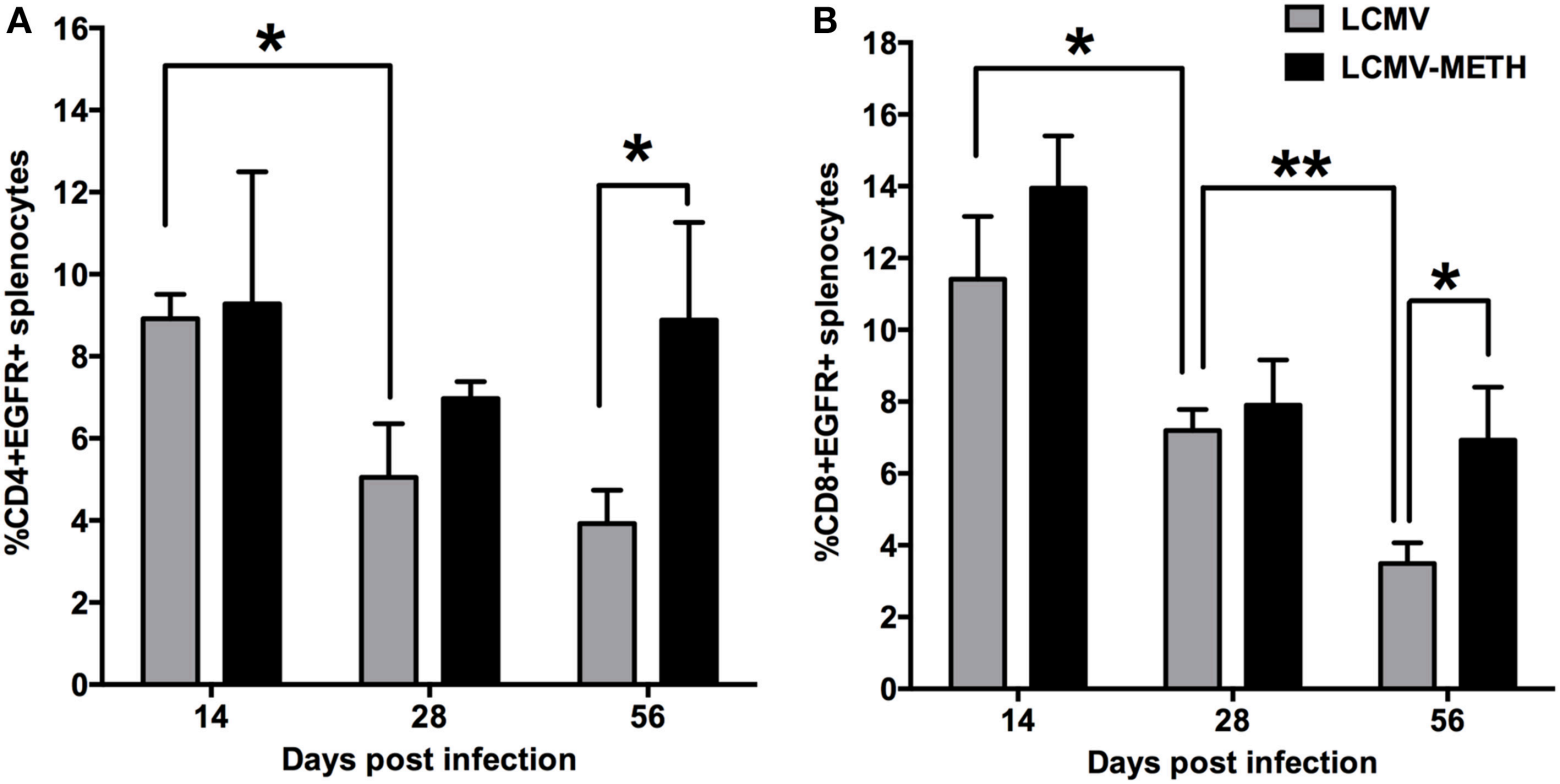
**Chronic METH treatment increases EGFR expression on splenic T cell subsets in LCMV infection**. Splenocytes extracted from LCMV infected mice with or without METH treatment, were surface stained after antigen specific stimulation with CD3, CD8, CD4 and EGFR markers. FACS analysis was performed and analyzed as described in Methods section. METH significantly increased EGFR expression at later stages of infection both in CD4 **(A)** and CD8 **(B)** T cells. EGFR expression was significantly decreased during the course of infection both in CD4 and CD8 T cells; (^*^p < 0.05; ^**^p < 0.01).

In recent years, increasing evidence has shown that viruses can interact with and modulate epidermal growth factor receptor (EGFR) activity to facilitate viral entry, replication or the evasion of host immune surveillance (Zheng et al., 2014). Furthermore, EGFR is a receptor tyrosine kinase that regulates cellular homeostatic processes (Mendelsohn and Baselga, 2006). The responses that are induced by ligand binding to EGFR, including cell signaling activation, protein kinase phosphorylation, and cytoskeletal network rearrangement, resemble those induced by virus infection (Diehl and Schaal, 2013). Intriguingly CD4+ (Figure 6A) and CD8+ (Figure 6B) T cells showed a significant increase of EGFR expression in the METH exposed LCMV infected group than LCMV alone by 56 days post infection, while a trend of increased expression in the METH treated group was observed at all time-points analyzed (Figure 6). In LCMV infected mice alone, EGFR expression in both CD4+ and CD8+ T cells decreased in a time dependent manner. The difference in the expression reached significance only at day 56 because the viral infection *per se* steeply decreased EGFR expression. As EGFR has been shown to facilitate viral entry, this decrease in expression could be a way the host immune system is responding to evade further infection. This data is very interesting and clearly indicates that chronic METH exposure increases EGFR that probably increases permissiveness of viruses and be in favor of establishing infection. We are interested to pursue further the molecular aspects of this observation.

### LCMV induced cytokine profile is altered in the presence of METH

**Figure 7:**
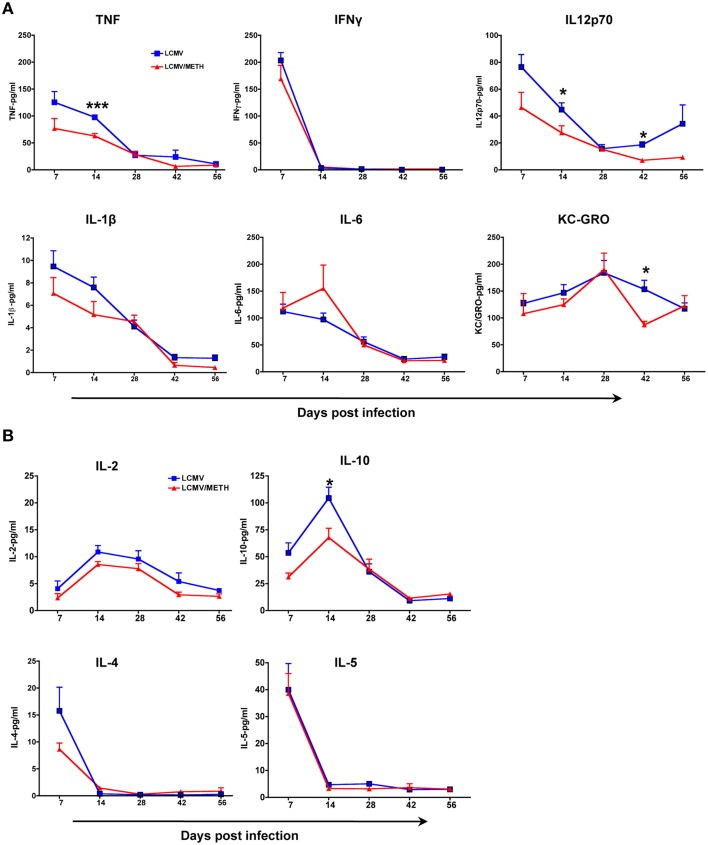
**LCMV induced cytokine profile is altered in the presence of METH**. Mouse cytokines in serum were measured using a Meso Scale Discovery (MSD) multiplex 7-spot electrochemiluminescence (ECL) assay and outputs measured by an ultra low noise charge-coupled device (CCD) Imager 2400 (Meso Scale Discovery, Gaithersburg, MD, USA). Sera was collected from the LCMV infected mice with or without METH exposure at days 7, 14, 28, 42, and 56 post-infection. **(A)** The proinflammatory cytokines measured included TNF, IFNγ, IL12p70, IL-1β, IL-6 and KC/GRO. **(B)** The cytokines measured to analyze the Th2 profile included IL-2, IL-10, IL-4 and IL-5 (^*^p < 0.05; ^***^p < 0.001). METH decreased IL-2 levels in the serum at all time-points analyzed; however the statistics did not reach significance.

Experimental data from numerous systems demonstrate that CD8 and CD4 T cell responses co-operate to control viral infections by production of cytokines as well as inducing target cell lysis. We analyzed the production of the signature proinflammatory (Figure 7A) and Th2 cytokine profile (Figure 7B) in the serum. LCMV infection induced a very good titer of all the major proinflammatory cytokines (TNF, IL12p70, IL1β, IL-6) by day 7 that gradually decreased as infection proceeded. METH significantly decreased the production of both the major proinflammatory cytokines, TNF, and IL-12 in serum by day 14 (Figure 7A). Although METH exposed LCMV infected mice showed decreased titers of the cytokines than LCMV alone, the differences did not reach significance as infection proceeded. IFNγ levels, however was very high at day 7 that drastically dropped to baseline by day 14 and not seen in circulation anymore until the end of the study. METH did not alter IFNγ levels at day 7 or any other time point. TNF is a very important proinflammatory cytokine and IL12 is an important cytokine that determines the Th1 fate. The other cytokines that we analyzed as part of the proinflammatory profile (IL1β, and KC/GRO) showed a decreased production in the METH treated group than without, although the values did not reach significance. KC/GRO is a CXC related protein that is mainly secreted by macrophages and is a chemoattractant for neutrophils (Shiratori et al., 1994). It is part of the proinflammatory profile that is altered in chronic viral infections.

We found that IL-2 levels in the serum was decreased at all time-points in the LCMV-METH group than LCMV alone (Figure 7B), in concordance with what we reported in human PBMCs (Potula et al., 2010). The regulatory cytokine IL-10 was also decreased upon METH exposure at early time-points, while levels of the Th2 cytokines, IL-4 and IL-5 remained unaltered. In all, the cytokine data indicate that METH profoundly affected the Th1/proinflammatory responses.

## Discussion

It is now well-established that METH abusers, as other drugs of abuse, are at increased risk of various infectious diseases. METH is typically administered nasally, intravenously or orally, and METH users experience feelings of euphoria, hyperactivity, reduced appetite, sleeplessness, and arousal after administration (Cruickshank and Dyer, 2009). Effects of METH on systemic immune responses have been typically studied using intraperitoneal or intravenous administration (Harms et al., 2012). In this report, we have analyzed the effects of METH upon subcutaneous (s.c.) administration, and show that METH profoundly affected the immune response in the chronic LCMV infection model.

Irrespective of the route of administration, METH is metabolized largely in the liver. Unfortunately we could not assess the levels of METH in the liver due to technical issues. Studies indicate that metabolism does not appear to be altered by chronic exposure (Volkow et al., 2010). The terminal plasma half-life of METH of approximately 10 h is similar across administration routes, but with substantial inter-individual variability. Our results show approximately 1000–2000 pg/μl of METH after 2 h of s.c. injections that is consistent with other studies with any other route of injection (Riviére et al., 2000). Also in our study, METH effects on body weight of the infected animals were mostly impacted during the early weeks, as the viral replication was also higher at this time. METH injection has been shown to cause approximately 2 g-weight loss in mice in a chronic administration study (Martinez et al., 2009). In another study, oral administration of amphetamines in mice revealed no difference in body weights between treatment and control groups (Kwack et al., 2014), indicating that METH effects on growth may depend on route of administration.

LCMV has proven to be a great model to study chronic infections in mice as they induce a robust T cell response. Our finding of a significant increase in LCMV viral loads at day 3 in the METH treated group is consistent with other reports showing that METH increases viral replication and so the increased viral load seen in circulation. Intriguingly the levels normalize as the infection proceeds, and with or without METH show similar viral loads even up to the end of the study (56 days) (Figure 2). METH effects did not impact viral loads in tissues in our model while in a HSV model a modest increase is shown in tissue in the presence of METH (Valencia et al., 2012).

Antigen specific CD8 T cells undergo rapid clonal expansion in response to infection with intracellular pathogens and differentiate into cytotoxic effector T cells that control the infection through lysis of the infected cells and production of cytokines (Williams and Bevan, 2007). CD4 Th cells are pivotal and orchestrate the immune response and differentially activate diverse branches of innate and adaptive immunity to direct the appropriate response to an invading pathogen. CD4 Th1 immunity is critical to sustain residual CD8 T-cell activity to control infection during persistent infection and is characterized in CD4 T cells by the secretion of IFN-γ, TNF-α, and IL-2 (Szabo et al., 2003; Ng et al., 2013). We did not find any dramatic alterations in the percentages of either total CD4+ or CD8+ T cells in the spleen upon chronic METH exposure in the spleen, suggesting that METH may not alter the total percentages of the cells but probably their functional capability. Alterations in the CD4+ and CD8+ T cells upon METH administration have been shown to be very variable upon different routes of administration. While the CD4+ T cells have been shown to be decreased and CD8+ to be increased in a rat self-administration model of METH (Mata et al., 2015), another study using oral route showed the opposite (In et al., 2005) and one that used intra peritoneal route showed decreased percentage of both CD4+ and CD8+ T cells (Harms et al., 2012). Nonetheless, we did see a significant increase in the total CD3+ T cells that probably indicates overall activation.

Exhaustion of CD8+ T cells, defined as the progressive loss of functions caused by ongoing antigen exposure and is a major factor leading to defective pathogen clearance in chronic viral infections. Studies in the murine LCMV model have identified PD-1 as a critical mediator of this immune impairment. Blockade of the PD-1 pathway is considered a promising approach in both infectious diseases and cancer (Porichis et al., 2011). Porichis et al. (2011) have demonstrated in HIV infected patients, that PD-1 impairs HIV-specific T helper responses both by limiting expansion of these cells and by inhibiting effector functions of multiple differentiated CD4 T-cell subsets and that blocking these responses can overcome these effects. We found METH significantly upregulated PD-1 on both CD4+ and CD8+ T cells in our chronic LCMV model, suggesting METH profoundly affected both CD4+ and CD8+ T cells to go into exhaustion. The expression of PD-1 at later stages of infection for both CD4+ and CD8+ T cells was similar between the METH treated or untreated infected mice. This may be because, as the infection progressed METH no longer influenced the already exhausted cells and therefore we did not see much variation in the percentages of cells expressing these markers, correlating with the trend of chronic infection (Petrovas et al., 2013).

One of the earliest classic cell surface antigens expressed by T cells following activation is CD69. The function of CD69 on T lymphocytes acting as a costimulatory molecule in proliferation and lymphokine secretion is well-established (Borrego et al., 1999). The kinetics of CD69 expression in both subsets of T cells decreased during the course of infection. The reasons for this dynamic regulation of CD69 are not clear but it is probable that there might be a period of time later during infection when T cells are refractory to further upregulation of CD69. By contrast, METH increased CD69 expression of CD8+ T cells at early infection indicating increased activation. It has also been shown that upregulation of CD69 expression inhibits lymphocyte migration and T cells remain at the site of infection (Feng et al., 2002; Shiow et al., 2006). METH may have an important impact on T cell migration and may alter further consequences in the immune response, including generation of T cell memory. However, expression of both CD25 and CD27 markers were not much affected by METH at any time point analyzed either on the CD8+ or CD4+ T cells. The expression of CD69 and CD25 has been found to be dissociated in various situations as for example in normal pregnancies where CD25 is actually decreased and CD69 is increased (Chao et al., 2002). We observed that CD25, the high affinity IL-2 receptor that is shown to be induced upon this cytokine (Raué et al., 2013), did not alter much in the splenic T cell populations, probably correlating with the decrease in IL-2 upon METH treatment and non-activation of this marker. CD27 belongs to the tumor necrosis factor receptor (TNFR) family and has also been used as a marker to identify memory precursor CD8+ T cells (Prasad et al., 1997; Joshi and Kaech, 2008). During acute (Armstrong) or chronic LCMV (clone 13) infection, CD27 initially increases and then returns to the baseline levels with T cells remaining CD27-positive throughout the infection (Welten et al., 2013; Mbanwi and Watts, 2014). Our observation in the LCMV infected group is similar to these reports.

A major impact of METH in our study, besides PD-1 upregulation, was the strong increase in the migratory chemokine marker CXCR3. Activated Th1 CD4 T cells and effector CD8 T cells (Moser and Loetscher, 2001; Hu et al., 2011) express this receptor, and promote migration of activated CD8 T cells into non-lymphoid tissue infection sites under the influence of the chemokine ligands CXCL9 and CXCL10 (Weng et al., 1998; Christensen et al., 2006). Proteomic analysis of immature dendritic cells revealed a very strong increase of CXCR3 in METH treated cells than controls, implicating the role of METH in regulating migration (Reynolds et al., 2007). In this study we did not see any huge differences in the CXCR3 expression on CD4 or CD8 T cells between control and METH in the uninfected group (data not shown). The upregulation of CXCR3 upon infection in a METH environment has a strong implication that METH can affect CD8 T cell responses as well as generation of memory response. Matloubian's group have demonstrated in a chronic LCMV infection model that absence of CXCR3 leads to generation of more long-lived memory CD8 T cells with a qualitatively better recall response(Hu et al., 2011), tying with our findings to suggest that increase in CXCR3 upon METH treatment may evoke a poor memory response.

EGFR has also been confirmed as a co-receptor for the entry of viruses such as human cytomegalovirus (HCMV) and adeno-associated virus serotype 6 (AAV6) (Weller et al., 2010; Diehl and Schaal, 2013; Zheng et al., 2014). In addition, EGFR-mediated different signaling cascades have also been implicated to facilitate viral replication or viral pathogenesis, such as EBV (Kung et al., 2011) and Pichindé virus (Bowick et al., 2007). With this background we aimed to test if METH modulated EGFR expression on LCMV infected T cells. The increased expression of EGFR in the METH treated infected animals at all time-points probably suggests that METH might help promote viral entry and we speculate that upon EGFR signaling may also impact viral pathogenesis. In this study, the persistent elevated expression of EGFR observed in chronically infected mice exposed to METH compared to infection alone is worth noting. As more evidence are accumulating (Weller et al., 2010; Kung et al., 2011; Zheng et al., 2014) showing the importance of EGFR on viral entry and maybe pathogenesis, this interesting phenomenon certainly warrants further investigation at the molecular level. It will be interesting to determine the relationship between EGFR expression and PD-L1/PD-1 axis (Chen et al., 2015) in T cells in a chronic infection setting under the influence of stimulant-induced microenvironment.

While we saw interesting alterations caused by METH exposure in many of the important markers induced during chronic LCMV infection in the spleen, due to technical problems, we could not assess the impact on immune cells in the blood, which probably could have explained if this pattern is specific to secondary lymphoid organs as spleen. Nonetheless, changes in the cytokine profile in serum are representative of some of the effects of METH during the entire course of infection.

Chronic METH exposure via s.c. route majorly resulted in a dampened proinflammatory cytokine profile upon infection. We did find suppressed IL-2 levels throughout the study consistent with our earlier report on decreased IL-2 levels in T cells upon METH treatment (Potula et al., 2010). Brooks et al. have shown that persistent viral infection in mice results in a significant upregulation of interleukin IL-10 by antigen-presenting cells, leading to impaired T-cell responses (Brooks et al., 2006; Wilson and Brooks, 2011). Intriguingly, METH at early time points decreased IL-10 in circulation with infection than METH untreated infected mice, but quickly normalized as infection progressed. The role of IL-10 as a regulatory cytokine is still complex (Wilson and Brooks, 2011). Analysis of METH effects on plasma cytokines after s.c. administration, in mice and of METH dependent human subjects, revealed a lot of variation in the expression of IL-2 and IL-10 in both human and mice (Loftis et al., 2011). Alterations in the cytokine profile by METH may also be tissue specific as indicated by a significant upregulation of cytokines in the hippocampal tissue in mice (Loftis et al., 2011).

Our study indicates that chronic METH exposure via s.c. route alters systemic and peripheral immune responses. METH modulates key markers on T cells involved in LCMV pathogenesis. These findings of increase in inhibitory phenotype, PD-1, indicates that METH enhances T cell exhaustion. The significant increase of CXCR3 upon chronic METH exposure suggests that METH may not only play an important role in recruitment of CD8 T cells to elicit immune response but also a key player in modulating the generation of memory response.

## Author contributions

All authors participated in the design of experiments, interpretation of data and writing of the manuscript. US, BH, JC, and LG performed the experiments and analyzed the data.

### Conflict of interest statement

The authors declare that the research was conducted in the absence of any commercial or financial relationships that could be construed as a potential conflict of interest.

## Supplementary material

Figure S1 **Splenocytes extracted from LCMV infected mice with or without METH treatment, were surface stained after antigen specific stimulation with CD3, CD8, CD4 along with activation markers**. FACS analysis was performed and analyzed as described in Materials and Methods Section. **(A)** Representative histograms of CD3, CD4, and CD8 expression are shown along with the isotype control staining: LCMV in blue; LCMV-METH in red and isotype in shaded gray. **(B)** Representative histograms of activation markers already gated on CD3 and analyzed in CD4 or CD8 subset. Isotypes for the respective antibodies are plotted in shaded gray for all activation markers.
