# Supplementary figures and images for: Methamphetamine mediates immune dysregulation in a murine model of chronic viral infection
Source: Front Microbiol. 2015 Aug 11;6:793. doi: 10.3389/fmicb.2015.00793 (PMC4531300; doi:10.3389/fmicb.2015.00793)

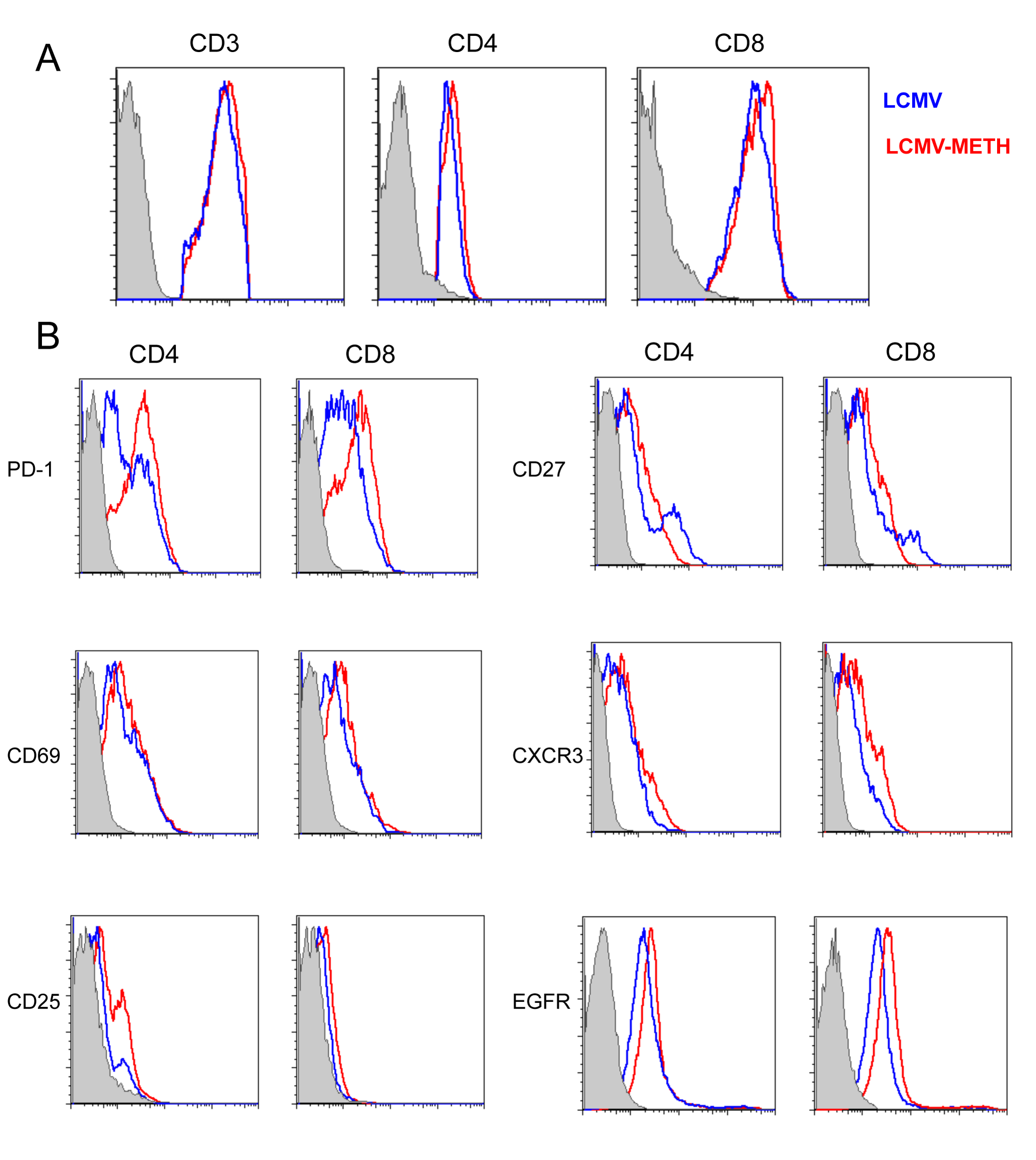

Supplement: Figure S1 — Splenocytes extracted from LCMV infected mice with or without METH treatment, were surface stained after antigen specific stimulation with CD3, CD8, CD4 along with activation markers. FACS analysis was performed and analyzed as described in Materials and Methods Section. (A) Representative histograms of CD3, CD4, and CD8 expression are shown along with the isotype control staining: LCMV in blue; LCMV-METH in red and isotype in shaded gray. (B) Representative histograms of activation markers already gated on CD3 and analyzed in CD4 or CD8 subset. Isotypes for the respective antibodies are plotted in shaded gray for all activation markers. [file Image1.TIF]
